# Ocular Manifestations of the Sturge–Weber Syndrome

**DOI:** 10.18502/jovr.v16i3.9438

**Published:** 2021-07-29

**Authors:** Kiana Hassanpour, Ramin Nourinia, Ebrahim Gerami, Ghavam Mahmoudi, Hamed Esfandiari

**Affiliations:** ^1^Ophthalmic Research Center, Research Institute for Ophthalmology and Vision Science, Shahid Beheshti University of Medical Sciences, Tehran, Iran; ^2^Department of Ophthalmology, Labbafinejad Medical Center, Shahid Beheshti University of Medical Sciences, Tehran, Iran; ^3^Department of Ophthalmology, Northwestern University Feinberg School of Medicine, Chicago, IL, USA

**Keywords:** Choroidal Hemangioma, Glaucoma, Ocular Manifestations, Sturge-weber Syndrome

## Abstract

Sturge–Weber syndrome (SWS) or encephalotrigeminal angiomatosis is a non-inherited congenital disorder characterized by neurologic, skin, and ocular abnormalities. A somatic activating mutation (R183Q) in the *GNAQ* gene during early embryogenesis has been recently recognized as the etiology of vascular abnormalities in SWS. Approximately, half of the patients with SWS manifest ocular involvement including glaucoma as the most common ocular abnormality followed by choroidal hemangioma (CH). The underlying pathophysiology of glaucoma in SWS has not been completely understood yet. Early onset glaucoma comprising 60% of SWS glaucoma have lower success rates after medical and surgical treatments compared with primary congenital glaucoma. Primary angle surgery is associated with modest success in the early onset SWS glaucoma while the success rate significantly decreases in late onset glaucoma. Filtration surgery is associated with a higher risk of intraoperative and postoperative choroidal effusion and suprachoroidal hemorrhage. CH is reported in 40–50% of SWS patients. The goal of treatment in patients with CH is to induce involution of the hemangioma, with reduction of subretinal and intraretinal fluid and minimal damage to the neurosensory retina. The decision for treating diffuse CHs highly depends on the patient's visual acuity, the need for glaucoma surgery, the presence of subretinal fluid (SRF), its chronicity, and the potential for visual recovery.

##  INTRODUCTION

Sturge–Weber Syndrome (SWS) or encephalotrigeminal angiomatosis is a rare congenital disorder that mainly affects the brain, skin, and eyes. The complete spectrum of SWS is characterized by leptomeningeal hemangioma, facial angiomatosis or port-wine stain (PWS), and ocular abnormalities.^[1]^

Roach et al classified SWS into three types: Type 1 (the most common) includes leptomeningeal and facial angioma with or without glaucoma; Type 2 presents with facial angioma as the most prominent manifestation with or without glaucoma, but no brain involvement; Leptomeningeal angioma is the only manifestation of Type 3, the rarest type, that is frequently diagnosed by brain scans.^[2]^

SWS occurs sporadically with an incidence ranging from 1 in 20,000 to 50,000 live births. No race or gender predilection has been identified. Despite a few familial clusters, SWS is considered non-hereditary.^[3]^

Children with SWS usually present with PWS or nevous flammeus as a congenital birthmark. PWS occurs in 3 per 1,000 births as a red or pink macula on the forehead, frequently unilateral. However, only 5–15% of children with PWS show other features of SWS.^[4,5]^ V1 or V2 distribution of PWS, the original description of PWS, has been recently challenged by Waelchli et al.^[6]^ They proposed that facial involvement follows embryological vasculature rather than trigeminal nerve distribution.^[6]^

Neurologic signs and symptoms including seizure, headache, stroke-like episodes, hemiparesis, visual field deficits, and cognitive impairment variably present throughout life. Seizure is often the initial neurologic presentation in 80% of SWS patients, starting in the first year of life.^[7]^ Mental retardation is also a common neurologic feature.^[8,9]^

The eye is found to be affected in approximately half of the patients with SWS. In the present review, we aim to further discuss the ocular manifestations of SWS with emphasis on glaucoma and diffuse choroidal hemangioma (CH) as the two most common ocular complications.

##  METHODS

We searched Medline using PubMed and Google Scholar, reviewing articles published between 1970 and 2020. Our keywords included multiple combinations of “Sturge-Weber Syndrome”, “glaucoma”, “pathophysiology”, and “pathogenesis”. The following medical subject headings (MeSH) were also used.

• Sturge-Weber Syndrome/complications*

• Sturge-Weber Syndrome/physiopathology*

• Sturge-Weber Syndrome/surgery*

• Sturge-Weber Syndrome/therapy*

• Disease Management*

• Glaucoma*/diagnosis

• Glaucoma*/etiology

• Glaucoma*/therapy

• Humans

• Intraocular Pressure/physiology*

• Sturge Weber Syndrome

• Syndrome, Sturge-Weber

The articles were reviewed and only articles published in English language with available full-texts were included in the present study.

**Figure 1:**
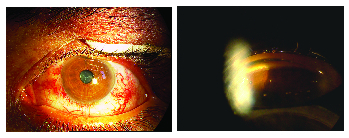
Left image shows conjunctival and episcleral abnormal tortuous vessels in a patient with Sturge-Weber syndrome. Presence of blood in the Schlemm's canal in gonioscopy of the same patient.

**Figure 2:**
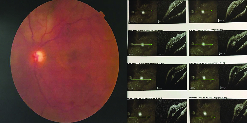
Left image shows diffuse choroidal hemangioma as diffuse dark and saturated red areas making “tomato-ketchup appearance” in the fundus of a patient with Sturge-Weber syndrome. Severe exudative retinal detachment is evident in optical coherence tomography images of the same patient.

### Pathophysiology of SWS

Most SWS manifestations result from vascular abnormalities. There are mainly two hypotheses proposed primarily to explain vascular malformations in SWS. The first hypothesis assumes a genetic mutation affecting vascular regulation during early embryogenesis, while the second hypothesis emphasizes on the role of a focal vascular dysplasia in the brain and subsequent involvement of the overlying eye and skin.

Shirly et al^[10]^ found a somatic activating mutation (R183Q) in the *GNAQ* gene which produces a protein (G alpha subunit q) regulating the signaling process that results in cell proliferation and inhibition of apoptosis. The mutation is also reported in the PWS blood vessels, either in isolation or associated with SWS.^[11,12,13]^ The sporadic inheritance of the disease supports the occurrence of a somatic mutation that does not affect the germline. The primitive vascular plexus enters the brain, skin, and eye in the first trimester.^[14]^ Therefore, somatic mutation within this interval correlates well with the clinical signs of SWS. Parsa et al proposed a local primary venous dysplasia in the brain as the primary insult. Consequently, collateral venous vessels transmit venous hypertension to the overlying eye and skin causing choroidal vascular anomalies and PWS, respectively.^[15]^ Parsa's hypothesis does not contradict the occurrence of a somatic mutation like *GNAQ*, however, it is unable to completely explain the bilateral PWS and absence of brain involvement in some patients with SWS.^[14]^

#### Ocular manifestations

A significant portion of the patients with SWS present with ocular involvement, especially glaucoma. Table 1 summarizes the ocular manifestations of SWS [Table 1].^[16]^

**Table 1:** Summary of ocular findings in Sturge-Weber syndrome^[16]^


**Orbital**	
General	
	Proptosis
	Lids
	Ptosis
	Port-wine birthmarks of eyelid
**Extraocular**	
Sclera	
	Nevoid marks or vascular dilation of the episclera
	Large, anomalous vessels in the episclera
	Dilation and tortuosity of episcleral vessels
	Episcleral hemangiomas
Conjunctiva	
	Conjunctival telangiectasia
	Conjunctival hemangiomas
	Dilation and tortuosity of conjunctival vessels
	Large, anomalous vessels in the conjunctiva
**Intraocular**	
Anterior Segment	
	Increased corneal diameter
	Iris discoloration
	Telangiectasia of the iris with heterochromia
	Dilation and tortuosity of the iris vessels
	Sluggish pupils
	Anisocoria or other disturbances in pupil reactions
	Deep anterior chamber angle
	Glaucoma
	Ectopia lentis
Choroid	
	Choroidal hemangioma
	Angioid streaks
Retina	
	Dilation and tortuosity of retinal vessels
	Retinal arteriovenous aneurysm
	Varicosity of retinal veins
	Glioma
	Retinal detachment
	Central retinal vein occlusion
Optic Nerve	
	Arteriovenous angioma
	Papilledema
	Optic atrophy
	Optic nerve cupping
	Optic nerve drusen
**Other**	
	Strabismus
	Nystagmus
	Loss of vision
	Cortical blindness
	Abnormal visual field due to the lesions in visual pathway
	Anisometropia

**Table 2:** Summary of important studies investigating different surgeries in the treatment of glaucoma associated with Sturge-Weber syndrome


**Author, year**	**Surgery**	**No. of eyes**	**No. of patients**	**Age at surgery (Mean ± SD, and/or range)**	**Outcome**	**Length of follow-up (Mean ± SD, and/or range)**
Wagner et al^[40]^ 1988	Trabeculotomy combined with cyclotherapy	6	5	Three weeks to nine years	All achieved controlled IOP Two patients needed additional surgery	Average 4.5 years From three to eight years
Iwach et al^[38]^	Goniotomy^[49]^ Trabeculotomy^[9]^ Trabeculectomy^[21]^	20 eyes Early onset 16 eyes late onset	30 patients	Not separately reported	Median stable time for goniotomy and medical therapy 101 months. For trabeculotomy 21 months For trabeculectomy 23 months	Mean 10 years Range: 2–21 years
Olsen et al^[39]^ 1998	Goniotomy Trabeculotomy	12 4	14	Mean 10 ± 4 months Zero days to four years	IOP ≤ 22 mm Hg in 66.7% of the eyes after one or more goniotomy or trabeculotomy	5.4 years Range: 1.4–15 years
Wu et al^[44]^ 2017	Trabeculotomy ab externo	34	32	Median (IQR) = 3 months (1.25, 6.75)	Cumulative proportion of overall success: 94.1%, 90.5%, 86.6%, 86.6%, and 86.6% at three months, six months, one year, two years, and three years, respectively	Median (IQR) = 15.5 months (9.50, 25.50)
Irkec et al^[37]^ 1999	Trabeculotomy and guarded filtration surgery	6	5	Between 23 days and 9 years	Lowered IOP in five eyes; two eyes needed additional medical therapy	6.3 Range: 2–11 years
Sood et al^[46]^ 2017	Combined trabeculotomy and trabeculectomy	22	20	Mean 18.64 ± 29.74 months 0.7–96 months	41.7% (10/24) of eyes qualified and modified qualified success No complete success	Mean SD 134.73 ± 67.77 months 61–288 months
Board and Shields^[48]^ (1981)	Combined trabeculotomy and trabeculectomy	5	5	Two months to fifteen years	Despite postoperative IOP control, it increased in three patients who had longer follow-ups No additional surgeries were needed	Median 11 months 6–36 months
Agarwal et al^[47]^ 1993	Combined trabeculotomy and trabeculectomy	18	16	Mean 17.8 months Range: Birth–7 years	( ≤ 22 mm Hg) in 11 eyes (61.1%)	42 months Range: 1–8 years
Mandal et al^[45]^ 1999	Combined trabeculotomy and trabeculectomy	10	9	1.5 ± 3.0 years Range: 1 month–9 years	All eyes maintained a postoperative IOP < 16 mmHg without medication	27.6 ± 16.4 months Range: 12–64 months
Ali et al^[49]^ 1990	Trabeculectomy	7	6	Mean 22.42 years Range: 7–38 years	Two eyes w/o meds Four eyes with meds One eye needed repeat trabeculectomy	Nine months to Nine years
Mohamed et al^[54]^ 2018	Trabeculectomy with MMC With Ologen	10 10	8 8	3–5 years	Complete and qualified success in 80% and 20% in MMC, 70% and 20% in Ologen	12 months
Hamush et al^[56]^ 1999	Ahmed glaucoma valve implantation	10	9	10 days–25.5 years Only three > 10 years	Cumulative probability of success of 79%, 59%, and 30% at 24, 42, and 60 months, respectively	Mean 910.5 days (SD 6 574.1 days)
Kaushik et al^[57]^ 2019	Ahmed glaucoma valve implantation	24	18	7.91 ± 5.02 Range: 1–15	Cumulative probability of success rate was 75%	2.12 ± 0.87 years
Budenz et al^[58]^ 2000	Baerveldt glaucoma implantation	10	9	Six weeks and thirteen years	All eyes had adequate IOP control ( ≤ 21 mmHg) without the need for additional glaucoma surgery	35 months Range: 10–50
Amini et al^[59]^ 2007	Molteno drainage device	9	7	9.6 +/– 3.7 years Range: 5–17 years	The cumulative probability of success was 97.2% at 12 months, 78.02% at 24 months, and 43.34% at the final follow-up	32 +/– 4.7 months Range: 20–36 months
**Author, year**	**Surgery**	**No. of eyes**	**No. of patients**	**Age at surgery (Mean ± SD, and/or range)**	**Outcome**	**Length of follow-up (Mean ± SD, and/or range)**
Audren et al^[55]^ 2006	Non-penetrating deep sclerectomy	9	9	Eleven days to twenty-four years	Success rates (including no need for anti-glaucoma medications) were 56%, 28%, and 0% at 6, 13, and 26 months post surgery	26.3 months Range: 6–48 months

**Table 3:** Summary of important studies investigating different treatment strategies in diffuse choroidal hemangioma associated with SWS. Only studies with more than three cases were included


**Author, year**	**Treatment modality**	**No. of eyes**	**Age at surgery (Mean ± SD, and/or range)**	**Outcome**
Randon et al^[76]^ 2018	External beam radiotherapy (20 Gy in 10 fractions)	26 eyes of 25 patients	Five years (4–41) years	Reduced tumor thickness (4.5 mm, 2.7 mm at the last visit) Resolved retinal detachment in all except two
Schilling et al^[77]^ 1999	External beam radiotherapy (20 Gy)	15 eyes of 12 patients with diffuse CH	18.3 years	Exudative RD resolution Shrinkage of the tumor was seen in five eyes
Arepalli et al^[82]^ 2013	Plaque brachytherapy (Iodine-125 plaque)	5 eyes	13 years Median 11 Range: 11–27 years	Complete regression of SRF in all cases
Zografos et al^[81]^ 1998	Proton beam radiotherapy	6 eyes with diffuse CH	Not reported	Resolution of exudative RD tumor regression

### Glaucoma

Glaucoma remains the most common ocular complication of SWS, which occurs in 30–70% of the patients. Glaucoma in SWS has a bimodal presentation: early onset glaucoma in 60% of the cases and late onset glaucoma in 40% of patients. The mechanism of early onset glaucoma is not determined yet but is attributed to abnormal angle development. While the appearance of the angle shares common abnormalities with primary congenital glaucoma including the anterior iris insertion to the trabecular meshwork (TM), direct attachment of the ciliary muscles to the TM rather than to the scleral spur, and increased opacification of tissues of the angle,^[17]^ the exact site of pathology is not identified. Some distinct characteristics of angle in SWS include flat iris insertion in some parts of the angle,^[18]^ prominent vascular loops at the iris root, and the presence of blood in Schlemm's canal [Figure 1].^[17,19]^ In a histopathologic study, Rosenbaum et al did not find any TM abnormality with light and electron microscopic examination but abnormal vessels were present in all of their four cases.^[20]^ The link between early onset glaucoma and SWS could be explained with the fact that SWS is primarily a vascular abnormality and there is a strong body of evidence that suggests SC and collector channels are developed from a venous plexus in early weeks of gestation.^[21]^ Therefore, it is plausible to think that the resistance to outflow in early onset glaucoma is located in the distal outflow pathway. The less than favorable response to angle surgery in these cases also supports more distal pathology rather than TM. There is not enough evidence regarding the role of elevated episcleral venous pressure (EVP) in early onset glaucoma; engorged vessels that are seen in adult onset glaucoma have not been reported in early onset glaucoma.

Since the collagen fibers are more elastic in early years of life, these children often develop secondary structural changes such as globe enlargement, tears in Descemet's membrane, and corneal edema.

Elevated EVP secondary to episcleral and choroidal vascular abnormalities contribute to late-onset glaucoma. EVP normally ranges between 8 and 10 mmHg. The exact relationship between chronic EVP rise and IOP is yet to be determined; however, chronically untreated elevated EVP can increase the IOP and remains the primary mechanism of glaucoma in etiologies like carotid-cavernous fistula, thyroid ophthalmopathy, and late-onset glaucoma in SWS. In support of this pathophysiology, Phelps et al^[18]^ investigated EVP in 12 patients with SWS. The mean EVP ( ± SD) was 18.1 ± 6.4 mm that was significantly higher than an average of 9.1 ± 1.6 mm Hg in the normal fellow eyes. Subsequently, Shiau et al^[22]^ confirmed their findings in 22 eyes with SWS. The possible role of SC and collector channels dysfunction which could potentially exacerbate the effect of high EVP in adult glaucoma has not been evaluated.

Accelerated aging of the angle structures has been reported in pathological investigations of late-onset glaucoma. First proposed by Cibis et al, a similar mechanism like primary open-angle glaucoma is also considered as the underlying mechanism.^[17]^ It is not clear if these changes are primary or develop in response to long-term high IOP.

Angle closure glaucoma has also been reported in SWS, although significantly less common than open-angle mechanism. Murayama et al^[23]^ reported a 14-year-old boy with unilateral acute angle closure glaucoma secondary to posterior scleritis in SWS. They postulated swelling of the ciliary body, choroidal effusion, anterior rotation of the ciliary processes, and swelling of the lens to be the underlying mechanism of angle closure. Ectopia lentis^[24]^ and angle neovascularization secondary to retinal pathologies^[25]^ have been reported to contribute to angle-closure glaucoma in SWS.

#### Diagnosis

All infants born with PWS should undergo thorough and frequent ophthalmic examinations. Repeat ophthalmic examinations every few months during the first years of life are recommended. IOP measurement in all sessions is mandatory even if the angle structure appears normal. If no sign of glaucoma is detected, an annual ophthalmic checkup is recommended.^[26]^

The risk of glaucoma is associated with the pattern of PWS and is estimated to be 25% with a large unilateral PWS and 35% with bilateral involvement.^[27,28]^ It is notable that nearly 40% of SWS patients have bilateral PWS.^[29]^

#### Glaucoma treatment

Glaucoma management in SWS is challenging and depends on the onset of the disease and the underlying pathophysiology. While medical therapy is usually tried as first-line therapy for early onset glaucoma, surgery is ultimately needed for the majority of the patients. Medical management remains the first choice in the late-onset glaucoma in SWS.

#### Medical treatment

Aqueous suppressants (beta-blockers and carbonic anhydrase inhibitors) and the outflow facilitators such as prostaglandin analogs have been successfully used. Agents lowering EVP are the potential future drugs that are not currently available.^[22]^ Awad et al^[30]^ reviewed 22 eyes with mostly early onset glaucoma (20 eyes) and found that antiglaucoma medications successfully controlled the IOP in only seven eyes of five patients within 62 months of follow-up. Van Emelen et al^[31]^ retrospectively reviewed the records of 19 SWS patients with the mean age of 8.2 years. Eight patients developed glaucoma (eight eyes with early onset glaucoma vs one eye with late-onset glaucoma). Seven out of eight patients (87.5%) treated by beta-blockers and carbonic anhydrase inhibitors needed glaucoma surgery to lower the IOP. Latanoprost is the most frequently investigated hypotensive agent.^[32,33,34]^ Yang et al^[34]^ used latanoprost in six SWS glaucoma with the age ranging from 4 to 19 years. Only two eyes that had juvenile onset glaucoma responded to latanoprost with a mean IOP reduction of 8.8 mm Hg. Similarly, Altuna et al^[32]^ investigated latanoprost in 18 SWS patients with the mean age of 19.7 ± 12.4 years and reported a success rate of 16.7% (3 of 18 patients) at the six-months follow-up. The causes for failure in this study were glaucoma surgery (seven patients), additional medical therapy (three patients), and intolerable conjunctival hyperemia (one patient).

In another study by Ong et al, 17 eyes with SWS glaucoma who started on latanoprost were retrospectively evaluated. The mean age of the patients at the onset of glaucoma diagnosis and the latanoprost start was 2.59 and 6.8 years, respectively. Fifty percent of the patients achieved successful outcomes after one year. However, treatment failure was reported as early as one month after the start of latanoprost in five (29.4%) patients.^[33]^

Wagnanski et al^[35]^ investigated the effect of oral propranolol (2 mg/kg) on IOP in four SWS infants with age ranging from 8 to 44 weeks. Despite IOP reduction at one week after the initiation of the treatment, IOP increased to an average of 20.7 (range 14–30) mmHg after one month. Subsequently, Kaushik et al^[36]^ used oral propranolol (2 mg/kg divided into two doses) one week before glaucoma surgery in SWS patients. These studies indicate that while oral propranolol temporarily lowers the IOP and can potentially be useful perioperatively, it is not suitable for long term in SWS glaucoma.

#### Surgical treatment

In glaucoma unresponsive to medical treatment, the choice of surgery needs to be decided on a case by case basis. Some surgeons prefer angle surgery as the primary operation in early onset glaucoma.^[39,40]^ While the exact pathology of early onset glaucoma in SWS is not determined, several studies reported favorable outcomes with goniotomy or trabeculotomy.^[37][38][39,40]^ Angle surgery removes the resistance at the level of TM and enhances the outflow through the physiologic pathways.

Studies investigating goniotomy or trabeculotomy as the initial surgical procedure report modest efficacy. Iwach et al^[38]^ reviewed angle surgery in 20 eyes with early onset and 16 eyes with late-onset SWS glaucoma. They reported the median stable time after a single goniotomy to be eight months in early onset glaucoma. However, it increased to nearly nine years after multiple goniotomies and adjunctive medical therapy. They found significantly shorter stable period in patients older than four years after goniotomy. Furthermore, trabeculectomy was performed in 21 eyes and achieved a stable duration interval with a median of 26 months. Additionally, 40% in the early onset and 17% in the late-onset group experienced choroidal effusion. Considering the higher rates of complications after trabeculectomy, the authors recommended goniotomy as the initial surgical choice in early onset SWS glaucoma. In another study by Olsen et al,^[39]^ 16 eyes of 14 patients with SWS glaucoma were studied. All patients were under four years of age. Twelve eyes underwent goniotomy while four eyes received trabeculotomy. Two-third of patients in the goniotomy group and half of the patients in the trabeculotomy group required a second procedure. One or more angle procedures resulted in IOP control in 66.7% of the patients after a median follow-up of 5.4 years. Wu et al^[41]^ retrospectively reviewed the clinical outcomes of trabeculutomy ab externo in 34 eyes with SWS glaucoma. The median age of patients at the time of surgery was three months. Complete (without any need for further glaucoma medications) and qualified (with the use of topical medications) success was achieved in 86.6% and 66%, respectively, with a median follow-up of 15.5 months. The authors concluded early diagnosis of glaucoma in SWS patients might lead to higher surgical success after trabeculotomy ab externo. Nevertheless, angle surgeries in SWS glaucoma achieve lower success rates compared with primary congenital glaucoma.^[42]^ Most individuals will need further surgeries or adjunctive medications to achieve the target IOP.^[43]^ Lower success rate of angle surgery in SWS indicates resistance in more distal outflow pathway. Wu et al^[44]^ showed that individuals with multiple episcleral vascular abnormal networks responded poorly to trabeculotomy compared to individuals with a simple episcleral vascular abnormal network. Their study supports a role for vessel-related factors in early onset glaucoma. Gonioscopy before any angle surgery is recommended in SWS to look for the landmarks of the angle as well as the presence of blood in the SC. The risk of intraoperative or postoperative hyphema varies between 25% and 88.2% after trabeculotomy in different studies.^[37,38,39,41]^

Combined trabeculotomy/trabeculectomy as the initial operation has been suggested in early onset glaucoma to address more distal resistance.^[45,46,47,48]^ Recently, Sood et al^[46]^ reported combined trabeculotomy/trabeculectomy in 24 eyes of 20 patients with SWS glaucoma with the overall success rate of 41.7% within 134.7 ± 67.7 months follow-up. Board and Shields investigated combined trabeculotomy/trabeculectomy in five SWS patients with the age ranging from 2 months to 15 years. Median length of follow-up was 11 months. Three patients with a longer duration of follow-up experienced IOP increase, however, none of the patients underwent additional surgeries. In another study, the records of combined trabeculotomy/trabeculectomy was reviewed in 10 eyes of which 9 had early onset SWS glaucoma with the mean age of 1.5 ± 3 years. Postoperative IOP remained < 16 mmHg in all patients within the mean follow-up period of 27.6 ± 16.4 months.^[45]^ Similarly, Agarwal et al^[47]^ investigated combined trabeculotomy/trabeculectomy in 18 eyes with SWS glaucoma. Eleven eyes (61.1%) had IOP ≤ 22 mm Hg after a mean follow-up of 42 months. Three patients underwent repeat surgeries.

Trabeculectomy remains an important surgical option in late-onset glaucoma associated with SWS. Ali et al^[49]^ reported favorable outcomes with trabeculectomy in six patients with late-onset glaucoma (mean age of 22.4 years) in nine months to nine years of follow-up. Nevertheless, four patients needed additional medical treatment and one patient underwent repeat trabeculectomy. In SWS patients, there is a serious risk of choroidal effusion or expulsive hemorrhages after trabeculectomy. Iwach et al^[38]^ reported intraoperative choroidal effusions in 24% of cases undergoing trabeculectomy including 40% of five cases in the early onset group and 17% of 12 cases of the late-onset group. Direct communication between the arteriolar system and choroidal vasculature, without any intervening vascular bed, results in a high choroidal vascular pressure. This high pressure is opposed by an increased IOP associated with increased EVP. When the IOP is reduced during the filtration surgery, the unopposed high choroidal vascular pressure causes choroidal effusion.^[38]^

The mechanism underlying choroidal effusion is similar to effusion formation observed with significant IOP reduction after glaucoma surgery, which results in rapid transudation of fluid from the intravascular spaces into the extravascular space.^[50]^ However, faster formation, more massive effusions, and higher prevalence are observed after filtration surgeries in patients with SWS.^[48,51]^ Accordingly, the presence of CH poses the eyes at a greater risk of both intraoperative or postoperative choroidal effusions. Pandey et al^[52]^ reported that 83.3% of the eyes that developed choroidal effusion had CH. Regardless of CH, higher choroidal vascular pressure and also vascular abnormalities such as abnormal vascular innervation^[7]^ and significant fragility^[51]^ make these patients more susceptible to choroidal effusion or hemorrhage.

To reduce the risk of choroidal effusion or expulsive hemorrhage, various modifications during surgery have been reported. Prophylactic posterior sclerotomy has been traditionally used by many surgeons.^[43]^ However, Eibschitz-Timoshi et al questioned the need for sclerotomy after investigating 17 eyes undergoing trabeculectomy.^[53]^ The authors reported no significant suprachoroidal hemorrhage and effusion in surgeries with modern techniques. Measurements such as generous use of viscoelastic devices for AC formation, maximum preoperative IOP control, prophylactic radiotherapy or laser photocoagulation of the CH are recommended to prevent the suprachoroidal hemorrhage.^[43]^

In a randomized clinical trial, Mohamed et al compared the outcomes of Mitomycin-C (MMC) augmented trabeculectomy and collagen matrix implant (Ologen) in the management of glaucoma in SWS.^[54]^ Twenty eyes of 16 SWS glaucoma patients with the age ranging from three to five years were divided into two groups. Complete and qualified success accounted for 80% and 20%, respectively, of patients in the MMC group after one year, while the corresponding values in the Ologen group were 70% and 20%, respectively. Complications in the MMC group included polycystic bleb in six patients, blebitis in one patient, and shallow anterior chamber in two eyes. Despite failure in 10%, the complication rate was minimal in the Ologen group. While physical spacers could improve the long-term outcomes of bleb function, these biodegradable implants could simultaneously act as physical resistance against overfiltration in the early postoperative days which is beneficial in offsetting suprachoroidal hemorrhage in SWS patients.

Non-penetrating surgeries like deep sclerectomy serve as alternatives to trabeculectomy. These modalities hypothetically lower the risk of choroidal effusion because of lower fluctuation of IOP during the procedures. Audren et al^[55]^ investigated non-penetrating deep sclerectomy in a series of nine eyes. The success rates without additional need for medical treatment were 56%, 28%, and 0% at 6, 13, and 26 months after surgery, respectively. However, two eyes developed choroidal effusion after NPDS.

Glaucoma drainage devices have been effectively used in SWS glaucoma. Hamush et al implanted Ahmad glaucoma valve (AGV) in 11 SWS glaucoma patients.^[56]^ The cumulative probability of success was 79%, 59%, and 30% at 24, 42, and 60 months, respectively. Similarly, Kaushik et al reported the results of AGV implantation in 24 eyes of 20 patients.^[57]^ The cumulative probability of success was 75% with a mean follow-up of 2.12 ± 0.87 years. Reported complications included intraoperative hyphema in four (16.67%) eyes, hypotony in three (12.5 %) eyes, and choroidal detachment in three eyes. Budenz et al studied two-staged Baerveldt glaucoma implantation in 10 eyes of nine patients and reported adequate IOP control in all operated eyes ( ≤ 21 mmHg) without the need for additional glaucoma surgery.^[58]^

Amini et al investigated Molteno implantation in nine eyes of seven patients.^[59]^ The cumulative probability of relative success was 97.2% at 12 months, 78.02% at 24 months, and 43.34% at the final follow-up. Postoperatively, massive choroidal effusion occurred in three patients which needed surgical drainage. Two eyes experienced bleb encapsulation and underwent needling bleb revision with 5-fluorouracil. Visually significant cataract and corneal endothelial touch prompting tube repositioning occurred in two patients, respectively.

Cyclodestructive procedures are usually limited to the eyes with low visual potential or eyes at higher risk of intraoperative complications.^[40]^ However, Van Emelen et al used it as the primary surgical option in seven eyes with SWS glaucoma.^[31]^ The authors found that cyclodestructive procedures reduced the IOP to < 22 mm Hg in six of the seven eyes with a mean duration of 4.5 years. The presence of episcleral hemangioma over the ciliary body makes the procedure more challenging.^[42]^

Table 2 summarizes various surgical treatments in the glaucoma associated with SWS.

Regardless of surgery types, patients need frequent and close follow-up examinations. Complications such as choroidal effusion and hypotony should be treated accordingly because chronically untreated choroidal effusions often lead to bleb failure or reduced visual acuity.

Laser treatment of facial PWS raised the concern of increasing IOP through the reduction of vascular channels serving to balance the venous pressure gradient.^[15]^ Sharan et al retrospectively compared 28 cases that underwent facial laser treatment and patients without laser treatment and did not find any difference between groups in terms of newly developed or exacerbating glaucoma.^[60]^

### Choroidal Hemangioma (CH)

CH, arising from choroidal vasculature, is a benign vascular tumor classified into two types of circumscribed and diffuse. Diffuse CH invariably correlates with SWS; patients with SWS often develop a diffuse type of CH. However, circumscribed CH has been occasionally reported in SWS.^[61,62]^ The prevalence of diffuse CH is 40–50% and is usually ipsilateral to the PWS.^[63]^

Diffuse CH is often detected during funduscopy as dark and saturated red area with the “tomato ketchup” appearance. Symptomatic patients present with reduced visual acuity, scotoma or flashing, refractive error, serous or exudative retinal detachment, macular edema, and retinal pigment epithelium (RPE) alterations with macular involvement.

Histopathologic investigations of diffuse CHs show some differences with circumscribed CHs and reveal containing considerable numbers of the enlarged pre-existing vessel and vascular channels lined with endothelium and complete lack of cellular proliferation of vessel walls. Areas of clustered vascular abnormalities mimicking circumscribed types have also been reported in diffuse hemangiomas.^[64]^

#### Diagnosis

Indirect ophthalmoscopy reveals the characteristic pattern of the fundus, so-called tomato ketchup appearance [Figure 2].^[65]^ Ultrasound, both B-scan and A-scan mode, is routinely used to confirm the hemangioma diagnosis in SWS patients. Diffuse thickening of the choroid in B-scan combined with high internal reflectivity of A-scan spikes confirms diffuse CH diagnosis.^[66]^ Fluorescein angiography, though difficult in children, shows rapid and speckled hyperfluorescent areas in the early phases due to the choroidal and vascular nature of the tumor. Similar to the circumscribed hemangiomas, though more widespread, staining with or without late leakage appears in the late phases.^[67]^ However, FA is more helpful in adults and patients with circumscribed CH. Indocyanine green (ICG) angiography shows the extension, vascularity, and arteriovenous shunts of choroidal changes; but this is an invasive diagnostic modality and may not be used in all cases especially in children.^[68]^

Recently, enhanced depth optical coherence tomography (EDI-OCT) has been employed in the diagnosis of CHs and following their response to treatment as well.^[69]^ Diffuse choroidal thickening in the involved eye and the fellow eye as well are observed by EDI-OCT.^[70]^ Surve et al investigated the role of swept-source optical coherence tomography (SS-OCT) in a large series of 34 eyes of 17 SWS patients.^[71]^ SS-OCT findings included loss of choroidal vascular pattern, increased choroidal thickness, and invisible sclerochoroidal interface. The authors reported a detection rate of 86.36% for SS-OCT compared with 50% clinically, 52.94% with FA, and 82.35% with ICG angiography. In addition, it has been shown that the outer retinal layers may be thinner in SWS patients with CH.^[72]^ A recent study reported small white dot-shaped “micro-drusen-like” changes of the retina in patients with diffuse CH.^[73]^

Furthermore, Griffith et al showed ocular enhancement in magnetic resonance imaging of SWS patients consistent with diffuse CHs.^[74]^ It has been shown that the CH on MRI images looks like sickle-shaped enhanced regions, thickest over the posterior portion of the globe and thinner toward the ciliary body.

The authors proposed MRI may play a substantial role in the diagnosis of diffuse CH. The advantage of MRI is that the children with SWS require neuroimaging irrespective of ocular findings that can be combined with ophthalmic sequences.^[75]^

#### Management

The goal of treatment in patients with CHs is to induce involution of the hemangioma, with reduction of subretinal and intraretinal fluid and minimal damage of neurosensory retina. The decision for treating diffuse CHs highly depends on the patients' visual acuity, the need for glaucoma surgeries, the presence of SRF and its chronicity and the visual recovery potential. Treatment of CHs may be difficult and therapeutic modalities may be limited because both juxtapapillary and foveal regions are often involved. The main risk of surgery in these cases is the increased risk of hemorrhage secondary to abnormal dilated episcleral and choroidal vasculature.

Various treatment modalities have been investigated including laser photocoagulation, plaque brachytherapy, external beam, proton beam, and stereotactic radiotherapy, photodynamic therapy (PDT), and anti-VEGF injections. The primary goal of treatment remains the resolution of SRF, however, treatment can also lead to the shrinkage of the CH mass.

Radiation-based modalities including external beam, plaque brachytherapy, proton beam, and stereotactic are rapidly expanding techniques. Randon et al investigated external beam radiotherapy (20 Gy in 10 fractions) in a large series (26 eyes of 25 patients) with diffuse CH.^[76]^ The treatment resulted in tumor regression; the mean thickness reached from 4.5 mm at baseline to 2.8 mm in the first year, and 2.7 mm at the last visit. The retinal detachment was resolved in all except two patients. Similarly, Schilling et al reported the result of low-dose radiation therapy in CH.^[77]^ Their study included 15 eyes of 12 patients with diffuse CH associated with SWS. The resolution of retinal detachment (in all patients) besides the shrinkage of the tumor (in five patients) was observed. Many other small case series reported the promising result of EBRT.^[78,79,80]^

Other techniques of radiation have been investigated in diffuse CH treatments. Proton beam radiation delivers an exact dose of radiation to a specific tissue. Zografos et al used the proton beam to treat six eyes with diffuse CH. They reported full resolution of the SRF in all of the treated eyes.^[81]^ Arepalli et al investigated plaque brachytherapy in five cases with diffuse CH associated with SWS.^[82]^ Complete regression of SRF was observed in all patients.

The main drawback of these technologies remains the high cost and unavailability in many hospitals. Furthermore, SRF generally resorbs slower after radiation (over several months) compared with PDT. Normal ocular tissues are also exposed to radiation doses with subsequent radiation-induced cataract, retinopathy, and optic neuropathy. However, the latter disadvantage occurs generally after EBRT and is reduced with other radiation-based treatments.

PDT allows for selective occlusion of vascular structures by photochemical destruction of vascular endothelial cells. PDT with verteporfin showed promising results for circumscribed CH. Correspondingly, multispot treatment has been applied in diffuse CH which causes the atrophy of the hemangioma vessels and reduces the leakage. Various isolated case reports used PDT and consistently reported complete resolution of SRF besides tumor involution.^[83,84,85,86,87]^ The main advantage of PDT includes the selective nature of the treatment preserving the overlying retinal vasculature and RPE. Currently, no significant complication has been reported with PDT. However, the studies investigating PDT in diffuse CH are usually small case series (to date < 15 cases) that make concluding difficult.

Some studies showed reduction of SRF after intravitreal injection of anti-VEGF agents.^[88]^ However, the continuous production of VEGF leads to unsuccessful long-term results with anti-VEGF therapy.^[89]^

Oral propranolol therapy was investigated in a few reports and revealed moderate effects in the resolution of exudative retinal detachment.^[90]^ The mechanisms which have been proposed for propranolol as a treatment modality for hemangioma can be classified into short-, mid-, and long-term effects. The most probable cause of the short-term effect is vasoconstriction. Blocking the release of proangiogenic factors such as VEGF, bFGF, MMP-2, and MMP-9 may lead to mid-term effects. The long-term effects may be related to the induction of apoptosis in endothelial cells and subsequent tumor regression.^[91,92]^ Serious side effects such as bradycardia and hypotension have been reported in cases using high-dose systemic propranolol. It has been shown that the intravitreal injection of propranolol leads to higher concentration of propranolol at the retinal level and lack of systemic side effects in comparison to the systemic usage of propranolol.^[93,94]^ The safe dose of intravitreal propranolol was determined in animal models and an experimental study showed that the intravitreal injection of propranolol was safe.^[95]^ Intravitreal propranolol has also been reported to be effective for the treatment of retinal angiomatosis.^[96]^ In addition, Nourinia et al successfully treated two cases of SWS with exudative retinal detachment secondary to diffuse CH with an intravitreal injection of propranolol (unpublished data).

### Other Ocular Abnormalities

PWS presents in the face of most patients with SWS. Vascular lesions on the eyelid, abnormal vascular lesions of the conjunctiva, episcleral hemangiomas, and dilated vessels of the retina are variably present in SWS patients and all are often ipsilateral to the PWS. Abnormal conjunctival vessels may be diffuse or localized. The diffuse type usually makes a pink or red hue in the affected eye. Iris heterochromia may also be present and is interestingly associated with a higher risk (45%) of ipsilateral glaucoma development.^[16,97]^ Ocular melanocytosis and iris mammillations have also been reported in SWS.^[61,98]^ Combined cilioretinal artery occlusion and hemiretinal vein occlusion was reported in a nine-year old boy diagnosed with SWS.^[99]^

Visual acuity results of 25 studies and visual field results of 12 studies were systematically reviewed to analyze the visual outcome of patients with SWS. The authors reported that VA was significantly reduced in 28% of eyes with glaucoma and 67% of eyes with diffuse CH. However, 70% of total SWS patients have normal visual acuity. Homonymous hemianopia was the most commonly reported visual field defect and was present in approximately 40% of the patients.^[100]^

##  SUMMARY

As the SWS mainly affects the brain, skin, and the eyes, a multidisciplinary approach including the neurologic, ophthalmic, and dermatologic evaluations are essential in the management of the disease. All involved specialists may face challenges in diagnosis, treatment, and determining the prognosis in patients with SWS. Considering the rare nature of the disease, most studies focusing on SWS are small case series.

Currently, most patients with glaucoma associated with SWS need surgical management. Since the lower success rates are achieved in these patients, the adjunctive medical therapy and the need for repeat surgeries should also be considered. The elucidation of the exact mechanism of glaucoma as the most common ocular complication can help better control of the disease.

Elevated EVP as a possible mechanism in the early onset glaucoma needs more clinical and experimental investigations. If the role of elevated EVP is further recognized, combined trabeculotomy and trabeculectomy can serve as the initial surgical procedure in the early onset glaucoma. Moreover, medical interventions targeting the EVP may have promising results.

The effect of SWS on the quality of life has been investigated with emphasis on neurological signs and symptoms^[101]^ but the possible effect of ocular complications on the quality of life of these patients has not been evaluated. Furthermore, the discovery of genetic mutations can be helpful in diagnosis and management of SWS.^[102]^ Further recognition of mutations, the possible molecular and cellular interactions and their downstream proteins can be targeted as promising treatments.^[103]^ Biomarker development to detect the association between clinical symptoms and disease prognosis is another area of research. Ocular involvement mostly diagnosed by optical coherence tomography will be a part of the future biomarkers.^[104]^

##  Financial Support and Sponsorship

Nil.

##  Conflicts of Interest

The authors do not have any conflicts of interest.
